# Drug-Modified Contact Lenses—Properties, Release Kinetics, and Stability of Active Substances with Particular Emphasis on Cyclosporine A: A Review

**DOI:** 10.3390/molecules29112609

**Published:** 2024-06-01

**Authors:** Iwona Rykowska, Ola Michałkiewicz, Iwona Nowak, Rafał Nowak

**Affiliations:** 1 Faculty of Chemistry, Adam Mickiewicz University, Uniwersytetu Poznanskiego 8, 61-614 Poznan, Poland; obstiwo@amu.edu.pl (I.R.); grzesiw@amu.edu.pl (I.N.); 2 Department of Ophthalmology, Military Institute of Medicine, ul. Szaserów 128, 04-141 Warsaw, Poland; raf.nowak@wp.pl

**Keywords:** therapeutic contact lenses, polymer matrix, drug stability, mechanic parameters, Cyclosporine A stability, drug delivery systems

## Abstract

The following review focuses on the manufacturing and parameterizing of ocular drug delivery systems (DDS) using polymeric materials to create soft contact lenses. It discusses the types of drugs embedded into contact lenses, the various polymeric materials used in their production, methods for assessing the mechanical properties of polymers, and techniques for studying drug release kinetics. The article also explores strategies for investigating the stability of active substances released from contact lenses. It specifically emphasizes the production of soft contact lenses modified with Cyclosporine A (CyA) for the topical treatment of specific ocular conditions. The review pays attention to methods for monitoring the stability of Cyclosporine A within the discussed DDS, as well as investigating the influence of polymer matrix type on the stability and release of CyA.

## 1. Introduction

Due to the complex structure of the eye, the lipophilic nature of the corneal epithelium, defence mechanisms, drug binding with tear proteins, enzymatic breakdown, and metabolism, traditional eye medications often suffer from low bioavailability and potential side effects. Furthermore, many patients, particularly the elderly, struggle to apply eye drops correctly, which can decrease the drug’s effectiveness and increase the risk of contaminating a bottle used over time.

Topical drug delivery is the most widely preferred route of drug administration to treat ophthalmic diseases such as keratitis, conjunctivitis, dry eye disease, glaucoma, and uveitis. Traditional ophthalmic drugs come in manifold forms; approximately 90% are administered as eye drops [1].

To address the limitations of traditional ophthalmic drugs, researchers are working on creating new and improved drug formulations for the eyes. These formulations aim to have longer residence times, high bioavailability, and controlled drug delivery to enhance safety and biocompatibility and reduce side effects. Additionally, the goal is to develop drug delivery systems that provide extended drug release at a therapeutic rate while maintaining pharmacokinetics and pharmacodynamics similar to eye drops. For these reasons, soft contact lenses (SCLs) as drug carriers have attracted researchers’ attention. The aim is to create therapeutic soft contact lenses (TSCL) that achieve the following [2,3,4,5,6]:

- Ensure the delivery of a therapeutic concentration of the active substance uniformly and continuously and of a precise amount onto the surface of the eyeball;
- Continuously release drugs from lens to tear film;
- Deliver medications with no adverse effect on the eye’s homeostasis;
- Maintain CL’s corrective parameters without loss and without disturbing the vision process;
- Are safe, simple, and eagerly used by patients.

Selecting suitable monomers and their mutual ratios determines the optical and mechanical properties and the potential application of polymeric matrices as drug carriers. By properly designing the composition of the matrix, one can ensure the stability of the drug substance, design the elution process of the drug, and tailor it to the needs of the patient. This is because the type of matrix used affects the chemical interactions present in the polymer structure, including the ability of the matrix to bind to the drug.

Over the years, techniques for modifying polymeric materials with active substances have changed rapidly from the simplest immersion methods to procedures drawing on advances in molecular, nano, and supercritical-fluid technologies [7]. To improve the drug loading capacity and prolong the drug release, several innovations have been made in the lens production process, such as the following:

- Drug application supported by the diffusion barrier created by vitamin E (VE) [8,9];
- pH-triggered contact lenses with CyA [10,11];
- Use of drugs in various systems and forms, including the following:
  - Cyclodextrin inclusion complexes [12];
  - Colloidal nanoparticles [13,14];
  - Nano- and microemulsions [15,16,17];
  - Micellar systems [18,19,20,21,22];
  - Liposomes [23,24].

**Figure 1:**
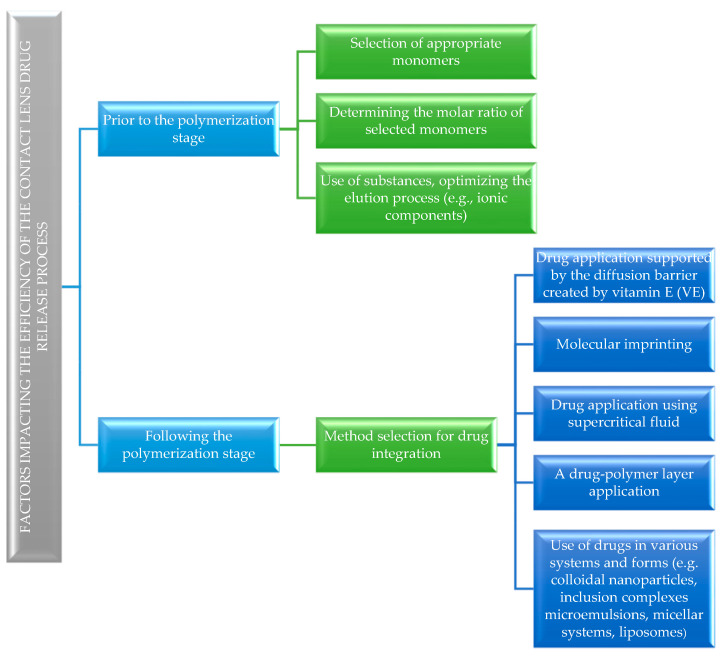
Factors affecting the drug release process.

Figure 1 illustrates factors affecting the efficiency of the drug release process from the polymeric drug delivery systems.

Conventional formulations of ophthalmic drugs, such as drops, ointments, or suspensions, typically afford only a 5% bioavailability of the active ingredient [25]. This results in 95% of the drugs present in the solutions being discharged from the surface of the eyeball, imposing the need to administer a large volume of solution (30 µL) [26] in a fivefold increase in the volume of the tear film (7 µL) [27].

The difficulty in topical eye treatment primarily stems from biological barriers and protective mechanisms that hinder the delivery of drugs to the eye. These barriers encompass the blood–aqueous, blood–retinal, and tear drainage barriers. Consequently, drugs encounter limited penetration to the eye surface or get washed away by tears into the nasal cavity and pharynx, potentially leading to systemic side effects [7,25,28].

According to mathematical modelling, contact lenses enable a minimum bioavailability of the active substance of 50%, in contrast to commonly used ophthalmic formulations, which constitute over 90% of the products available on the market [29]. This is attributable to a notable prolongation in the active substance’s residence time on the eyeball’s surface. In conventional formulations, this period can stretch to five minutes. However, within the lens polymer matrix, it may take several hours [30,31]. Other benefits of using TSCL include the following:

- The reduction in the administered drug dosage, mitigating undesired side effects;
- The potential for integrated therapy;
- The simplicity of use;
- Dosage personalization;
- No abrupt concentration changes;
- The potential for concurrent vision correction and pharmacotherapy.

Despite many technological innovations in soft contact lens-based drug delivery systems, most are still limited to the laboratory level. Providing effective DDS based on soft contact lenses poses many challenges for its developers. These include ensuring drug compatibility, stability, and effectiveness throughout the overall process, from manufacturing to storage, transport, and patient usage. Thus, the manufacturing of TSLC is complex and requires careful consideration of many factors [32]:

- Limited range of drugs: not all medications can be effectively incorporated into contact lenses;
- Customization challenges: achieving the proper drug dosage and release rate profile is complex and requires individualized adjustments;
- Risk of adverse reactions: some individuals may experience allergic reactions or other side effects;
- Irritation and discomfort: embedded drugs may cause irritation or pain in individuals;
- Sterilization and storage issues;
- Cost: drug-modified contact lenses can be more expensive than standard ones, making them less accessible to some individuals.

## 2. Cyclosporine A

Cyclosporine A is a well-known immunosuppressive agent composed of a cyclic undecapeptide with a molecular weight of 1203 Da and an octanol/water partition coefficient (Log Po/w) of 2.92 [33]. It is a highly potent and hydrophobic calcineurin inhibitor that hinders T-cell activation. CyA is used topically in eye inflammatory conditions like uveitis, corneal healing, and dry eye disease. Dry eye disease is a chronic illness characterized by symptoms of ocular discomfort and visual dysfunction resulting from abnormal tear quantity, quality, or fluid dynamics.

Difficulties encountered in the process of designing CyA-modified contact lenses include the following:

- CyA poor water solubility (0.012 mg/mL at 25 °C): CyA is a very hydrophobic drug (log *P* of 8.2);
- Chemical incompatibility;
- The rigid structure of the CyA molecule;
- Controlled release difficulties: controlled release of Cyclosporine A from a polymer material is challenging due to differences in diffusion rate and solubility;
- Possible interactions with the environment: Cyclosporine A can be sensitive to environmental conditions, which can lead to a change in its properties in the polymer material depending on storage and use conditions.

This review summarizes the latest developments in therapeutic soft contact lenses, mainly focusing on contact lenses modified with CyA, an immunosuppressive drug used topically in ophthalmology.

## 3. The Mechanism of Drug Transport

The release of the active ingredient from drug-modified lens matrices can transpire through various mechanisms, including diffusion, erosion, and biodegradation of the lens polymer matrix [34].

**Figure 2:**
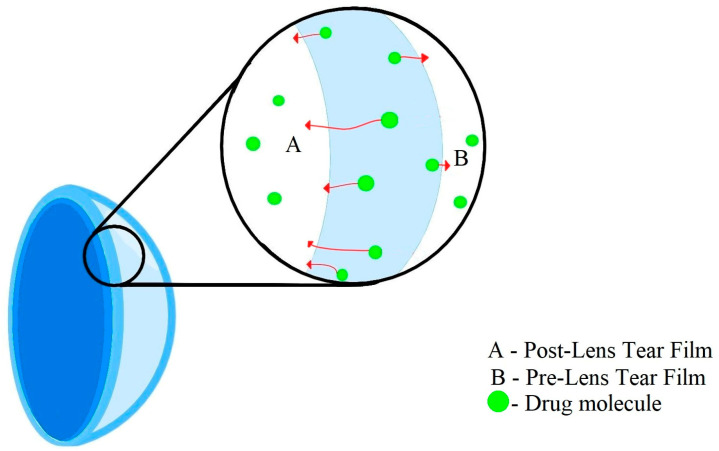
Diffusion process.

Diffusion (Figure 2) is a process in which, after placing a contact lens on the surface of the eyeball, drug molecules slowly migrate from the polymer matrix to the tear fluid, where the concentration of the substance is low (driven by the chemical potential gradient) [35,36]. This layer of tear fluid is referred to as the Post-Lens Tear Film (POLTF). It is located between the applied lens and the cornea. Its location directly affects the extension of the residence time of therapeutic substances on the eye’s surface. This is the result of limited mixing of tear fluid from the layer in front of the lens (Pre-Lens Tear Film, PLTF) and the layer behind the lens (Post-Lens Tear Film) [37,38,39].

Diffusion is the driving force of loading and releasing drugs from the water channels of contact lens matrices [40]. Its course depends on many factors, such as the water content, the thickness of the CLs, the properties of the applied drug molecules (molecular weight, solubility), and the time of application and modification of polymer surfaces with agents that slow down the elution of active substances [41,42].

Drug release through polymer matrix biodegradation is among several degradation processes polymers may undergo, including photo, mechanical, thermal, and chemical degradation [43,44].

Biodegradation is pivotal in controlled drug release systems, facilitating the gradual release of active substances from the polymer matrix. This process, influenced by molecular diffusion and polymer degradation, is directly impacted by the composition of the biodegradable polymer [45]

Biodegradable contact lenses comprise biocompatible polymers. Through degradation, lenses are solubilized in vivo, or non-toxic byproducts are released, allowing for safe elimination by the body without compromising its homeostasis [46].

**Figure 3:**
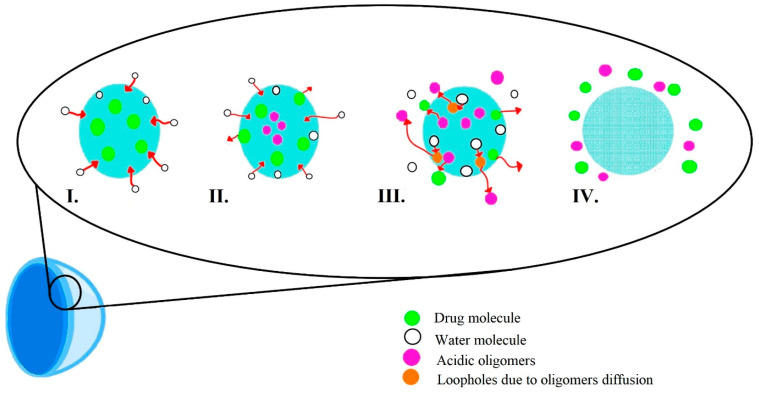
Polylactide/glycolide biodegradation process.

The biodegradation process varies according to the composition of the contact lens matrix. For instance, within the polylactide/glycolide group, four stages of degradation are delineated, as depicted in Figure 3.

The stages of hydrolytic degradation of the polymer matrix [47] are as follows:

- Water diffusion—diffusion of water molecules into the polymer matrix.
- Hydrolysis reaction—autocatalyzed hydrolysis reaction catalyzed by oligomer molecules with acidic end groups.
- Attainment of critical molecular weight—exchange of acidic oligomers from the lens matrix for water molecules, diffusion mechanism.
- The increase in porosity of the polymer matrix—increasing polymer matrix porosity slows degradation and stabilizes drug molecule elution.

Besides biodegradation, erosion also facilitates drug release. These processes often blur boundaries. Biodegradation involves the cleavage of hydrolytic bonds, producing soluble degradation products that can erode the polymer matrix when dissolved in water. Hence, biodegradation significantly contributes to erosion [48,49,50,51].

**Figure 4:**
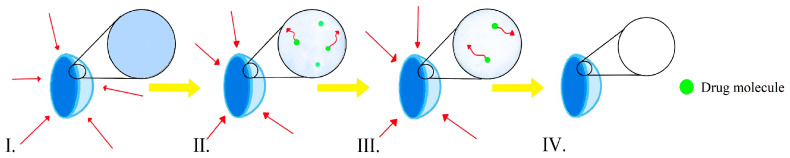
Bulk erosion.

There are two main types of erosion: surface and bulk erosion [52]. Bulk erosion transpires when water infiltrates the polymer matrix more rapidly than degradation. Consequently, polymer degradation proceeds uniformly across the lens matrix [53]. This phenomenon has been presented in Figure 4.

**Figure 5:**
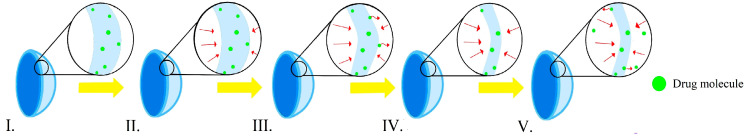
Surface erosion.

Conversely, when matrix degradation outpaces polymer infiltration by water molecules, the erosion process lacks uniformity within the drug delivery system (DDS) [54]. In such instances, surface erosion predominantly arises at the lens’s peripheries (Figure 5).

## 4. Materials for Contact Lenses

### 4.1. Polymers

The polymers incorporated into contact lenses can be categorized as biodegradable or non-biodegradable. Table 1 and Table 2 delineate these categories.

### 4.2. Contact Lenses’ Mechanical Parameters

**Figure 6:**
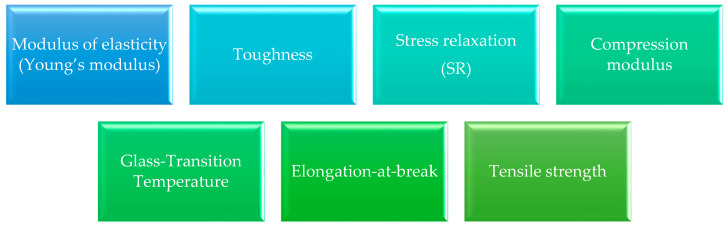
Mechanical parameters of contact lenses.

Contact lenses must meet several criteria to assess their suitability for use. These include mechanical parameters affecting optical properties and patient comfort (Figure 6).

- *Modulus of elasticity (Young’s modulus)*

This is a measure of contact lenses’ polymer stiffness, flexibility, or resistance to deformation [84]. It represents the ratio of stress to strain (load per unit cross-sectional area to local elongation) [85]. The higher the value of Young’s modulus, the harder the tested material is.

- *Toughness*

Polymer characteristics related to material plasticity are its toughness. The polymer characterized by toughness shows resistance to impact load and plastic deformation without damage to the material [86]. Toughness can be determined from the generated stress–strain curve by the ratio of energy break to the original sample volume [87].

- *Stress relaxation (SR)*

Unfavourable phenomena occur in the polymer network due to temperature, time, and environment. As a result, the structure may undergo stress relaxation, losing the initial stress, leading to matrix polymer failure at a critical moment [88]. Physical and chemical factors can trigger it.

- *Compression modulus*

This is a measure of the relative hardness of soft contact lenses. It is the ratio of the force (stress) necessary to deform the polymer structure by a given value [89].

- *Glass-transition temperature*

According to Bicerano, “the glass-transition temperature is the temperature at which the forces holding the distinct components of an amorphous solid together are overcome by thermally induced motions within the time scale of the experiment so that these components can undergo large-scale molecular motions on this time scale, limited mainly by the inherent resistance of each component to such flow” [90].

- *Elongation at break*

This is the ratio of the difference between the polymer’s final length and initial length to the initial length, subjected to stretching, expressed in percentage [66].

- *Tensile strength*

Strength acting per unit cross-section of the polymer at the critical point (failure) of the sample [89], expressed in Mega Pascals (MPa).

### 4.3. Techniques Used to Test the Mechanical Parameters of Contact Lenses

The mechanical parameters of contact lenses are examined using various techniques. Table 3 outlines these techniques, accompanied by concise descriptions and measurement techniques according to literature sources.

Separate criteria for determining the physical compatibility of contact lens care products with contact lenses are included and characterized in the ISO 18 369 standard [95]. These include the following:

- Diameter (hydrogel lenses only);
- Curvature (rigid lenses only);
- Back vortex power (all materials);
- Particular transmittance (among others, UV-absorbing lenses only);
- Physical appearance (e.g., colour, defects).

## 5. Polymer Matrix Impact on CyA Stability in Ophthalmic Applications

Ensuring the sustainable, effective, and safe delivery of the active substance from the polymer matrix is fundamental to the design of ophthalmic drug delivery systems. In this case, designing a process and drug formulation that ensures the stability of the active substance is crucial. Several key factors influence drug stability, such as the following:

- The drug-modified polymer matrix preparation process;
- Excipients: interactions with other formulation components, such as solvents or impurities, can lower the stability of active substances;
- Temperature: high temperatures can accelerate active substances’ chemical and physical degradation;
- Moisture can lead to hydrolysis or changes in the crystalline structure, reducing biological activity or physical stability;
- UV and visible radiation can cause the photodegradation of active substances;
- The environment’s uncontrolled pH value can affect the stability of drugs prone to acid or base hydrolysis;
- Oxygen: this can lead to the oxidation of oxidation-sensitive active substances.

Optimizing the process is crucial for maintaining the active substance’s stability and ensuring the drug’s efficacy and safety. This meticulous approach can be achieved through several methods, such as adding stabilizing additives like antioxidants (e.g., vitamin E) [3,96,97], maintaining a stable environment with pH buffers and stabilizers, or conducting the process in an inert atmosphere like nitrogen.

The following optimized drug modification procedures for various polymer matrices have been well documented in the literature [7];

- Drug application supported by inclusion complexes based on cyclodextrins (CD);
  ○ Copolymerization of the drug with cyclodextrin acrylic/vinyl derivatives;
  ○ CD implantation in polymer matrices;
  ○ Directed CD cross-linking on a polymer matrix;
- Drug application using colloidal nanoparticles;
- Drug application using polymeric nanoparticles (Polymeric Nanoparticles, PN);
- Drug application using liposomes;
- Drug application in micellar systems;
- Applying the drug in the form of a microemulsion;
- Deposition of a thin drug-polymer layer;
- Molecular printing.

Table 4 presents the research findings from studies on developing drug delivery systems containing Cyclosporine A for both commercial and laboratory contact lenses.

## 6. CL Modifications Techniques

Ensuring the effective and safe delivery of active ingredients presents a pharmacological challenge across various medical domains.

In ophthalmology, additional complexities arise, such as ensuring minimal impact on visual quality by the drug, maintaining a consistent and controlled dosage of the active substance delivered to the eye, and often encountering low drug bioavailability; consequently, the extensive research on modifying conventional methods of introducing active substances into the eye is understandable.

Table 5 summarizes this subject’s literature data and gives brief descriptions.

## 7. Methods for Investigating the Kinetics of Drug Release

Controlled-release drug delivery systems provide safe and therapeutic doses of the active ingredient to the target site until resources are depleted. It is hypothesized that the initial dose of the active ingredient may be higher to achieve a substantial initial drug concentration. Subsequent elution stages may deliver lower doses to maintain drug concentrations within the therapeutic range.

As the literature sources indicate, the most favourable drug release profile is the zero release profile [123,124]. However, after years of DDS research, it is stated that zero-order kinetics is not mandatory. The primary role of drug delivery systems is to ensure the drug concentration is within the therapeutic index (TI) range [124]. The TI is the ratio of the maximum safe drug concentration ($C_{max}$) to the lowest effective concentration ($C_{min}$) and is specific to the active substance [124,125].

To develop safe and efficient drug delivery systems (DDSs), it is essential to achieve controlled release kinetics of the active ingredient. Mathematical modelling is often employed for this purpose [126,127,128]. However, it demands a thorough comprehension of the drug release process and the consideration of all critical factors influencing drug elution.

**Figure 7:**
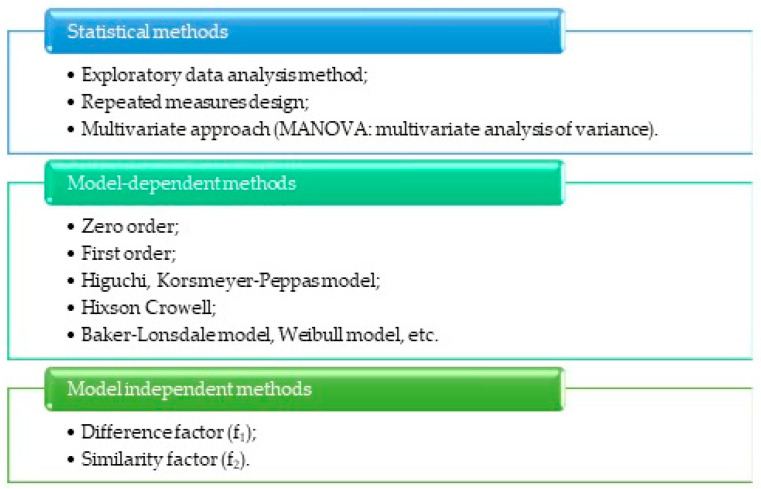
Approaches to assessing drug release kinetics from controlled drug delivery systems” [129,130].

Various approaches are recommended to investigate the kinetics of active substance release from the DDS. As depicted in Figure 7, these approaches are categorized into three groups.

## 8. Methods for Assessing the Stability of Active Substances

Testing the stability of released active substances is a fundamental and routine procedure necessary to evaluate their potential. Stable preparations are safe and enable effective patient therapy. Stability tests of the active substances are gathered in Table 6**.**

According to the FDA guide, a stability-controlling method must meet the recommended criteria. The analytical techniques used need to be the following [139]:

- Validated,
- Accurate,
- Precise, especially in the environment of interfering sample components, impurities, and degradation products of drug molecules.

Conducting studies to separate and identify degradation products is essential to testing the stability of active substances. Cyclosporine A is a widely studied example of such a substance [140]. Table 7 summarizes the analytical tools used to assess the chemical stability of Cyclosporine A presented in the literature.

Given its economic viability and widespread availability, HPLC emerges as the predominant technique for evaluating the stability of numerous active substances, such as Cyclosporine A, as evidenced by the abovementioned list. While other analytical methods are documented in the literature for assessing the stability of different active substances, HPLC stands out for its prevalent utilization. These include the following [141]:

- Nuclear magnetic resonance (NMR);
- Accelerated solvent extraction (ASE);
- Low-pressure liquid chromatography (LPLC);
- Thin-layer chromatography (TLC);
- Gas chromatography-mass spectrometry (GC-MS);
- Liquid chromatography–mass spectrometry (LC-MS);
- Capillary electrophoresis-mass spectrometry (CE-MS);
- Liquid chromatography–nuclear magnetic resonance (LC-NMR);
- Liquid chromatography Fourier-transform infrared (LC-FTIR).

## 9. Summary

This review offers a comprehensive exploration of polymeric matrices employed as carriers in advanced ophthalmic drug delivery systems, focusing mainly on developing DDSs as reservoirs of the immunosuppressant CyA. An extensive examination of the contemporary literature delves into the diverse array of polymeric carriers utilized, categorizing them into biodegradable and non-biodegradable polymers. Additionally, we scrutinize factors influencing the efficacy of the drug release process, pivotal technical parameters ensuring the quality of modified polymers, and innovative technical solutions, such as methods for polymer modification and incorporating additives to enhance polymer loading and drug release efficiency.

The article offers comprehensive insights into various facets of stability testing for active substances, methodologies for their controlled release from polymer matrices, and techniques for evaluating drug stability and contact lenses’ mechanical properties. It specifically delves into applying Cyclosporine A in ophthalmic therapies, all within the purview of ISO 18369 standards.

Upon review of the existing literature, it can be asserted that using polymeric materials as carriers for active substances, including Cyclosporine A, represents a contemporary, secure, and practical approach to administering ocular medications. This method effectively aligns with the expectations of both healthcare providers and patients in the topical treatment of ocular diseases.

The authors of the referenced studies have demonstrated that modifying the application of active substances to polymer matrices, including the implementation of a diffusion barrier using vitamin E, the formation of inclusion complexes based on cyclodextrins, molecular imprinting, utilization of polymeric nanoparticles, microemulsions, micelles, incorporation of surfactants, employment of liposomes, and transferosomes, can have a positive impact on enhancing stability and extending drug delivery duration with DDS. It is crucial to select an appropriate method tailored to specific active substances and to optimize it to achieve optimal outcomes in ophthalmological therapies, ensuring safe drug concentrations for the patient.

As shown in the review, using contact lenses as carriers of active substances brings several benefits. Their potential is related to ensuring a balanced supply of the active substance, characterized by a release profile close to zero, as well as safety resulting from lower concentrations of drugs applied to the eyeball. In the case of many active substances, such as Cyclosporine A, this is of fundamental importance in minimizing the risk of undesirable side effects in internal organs.

TSCLs enable the supply of Cyclosporine A at all times after application, limiting the compound’s leaching from the surface of the eyeball. This plays an essential role in treating diseases such as dry eye syndrome, where the continuity of treatment allows the patient to increase their comfort and compliance and minimize corneal irritation.

Despite many years of efforts, it has not been possible to commercialize contact lenses modified with Cyclosporine A. Researchers still face many challenges and difficulties. These include the following:

- Selection of a carrier with optimal affinity for the drug (ensuring balanced drug release or use of controlled drug release triggers).
- Achieving an optimal drug release profile. The initial burst release must be overcome.
- Redetermination of the therapeutic dose of CyA administered by TSCL (taking into account greater availability of the drug, continuous supply of the drug, and the resulting risk of local and systemic toxicity or the risk of drug resistance.
- Maintaining the optical parameters of the matrix used.
- Maintaining the mechanical parameters of the lens (water content, flexibility, oxygen permeability [160]).
- Keeping the TSCL stable during storage, determining the expiration date.
- Maintaining the sterility of the device.
- Defining the shelf-life.
- Parameterization of the procedure on an industrial scale.
- Compliance with legislative requirements.

Progress on these challenges would greatly expand TSCL’s potential to deliver CyA at the desired rate and location in the eye.

The review demonstrates that techniques for creating intelligent drug delivery systems still need to be developed. The potential of systems based on polymeric materials remains untapped, and the kinetics of the delivery and release of active substances, including CyA, still need to be entirely satisfactory.

## Figures and Tables

**Table 1:** Biodegradable polymers.

Polymer	Characteristics	References
*Propoxylated glyceryl triacrylate (PGT)* 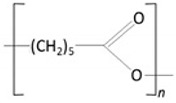	✓ Polymer with multiple vinyl functionalities; ✓ Used as nanoparticles that contain ophthalmological drugs inside; ✓ Used in Timolol-loaded PGT nanoparticles, tested on beagle dogs using commercially available Acuvue Oasys (Johnson & Johnson, New Brunswick, New Jersey) lenses.	[55,56,57]
*Chitosan (CT.)* 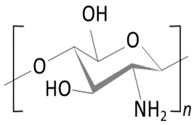	✓ Linear copolymer of β-(1–4) linked 2-acetamido-2-deoxy-β-D-glucopyranose and 2-amino-2-deoxy-β-D-glucopyranose;	[53]
	✓ Cationic polymer insoluble at high pH; at pH < 6 becomes water-soluble cationic polyelectrolyte;	[54]
	✓ Biocompatible; ✓ A broad spectrum of biological activity with high safety of use;	[55,56,58,59]
	✓ Extensively used for the delivery of CyA and pilocarpine.	[60]
*Poly-(lactic-co-glycolic acid) (PLGA)* 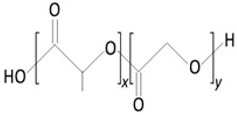	✓ Biocompatible; ✓ Utilized as nanoparticles containing ophthalmic drugs, peptides, proteins, and DNA;	[61,62]
	✓ Copolymer of polylactic acid (PLA) and polyglycolic acid (PGA); ✓ Widely favoured for its safe degradation, extensive clinical testing history, and capacity for effective and sustained drug delivery; ✓ Biodegradation products result from ester linkage hydrolysis in water; ✓ FDA approved;	[63]
	✓ Used in DDS based on hydrogels and nanoparticle production.	[64]
*Fibrin* 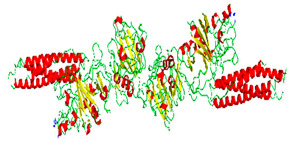	✓ Protein-based natural polymer produced from fibrinogen;	[65]
	✓ A fibrin sealant (FS) tested as a carrier of subconjunctival topotecan (TPT) in transgenic murine retinoblastoma (RB) treatment;	[66]
	✓ Tested as a drug delivery system as a liquid version (platelet-rich fibrin (PRF) composed of liquid fibrinogen/thrombin) for enhanced bone/cartilage tissue regeneration and as a subcutaneous implantation of discs under mouse skin remaining steady tetracycline (TET) to 12 days after application;	[67,68]
	✓ A fibrin sealant used as a reservoir of dexamethasone and methotrexate.	[69]
*Policaprolactone (PCL)* 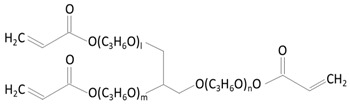	✓ Slow-degrading aliphatic polyester; ✓ Obtained from caprolactone; ✓ Has collagen-stimulating properties; ✓ Biocompatible, bioresorbable;	[70,71]
	✓ Approved by FDA; ✓ Used in producing SCL embedded with polycaprolactone-based nanoparticles.	[72,73]
*Poly (D, L-lactide)-dextran (Dex-b-PLA)* 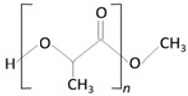	✓ Copolymer of poly(D, L-lactide) and dextran, self-assemble into core-shell structured nanoparticles, with size precisely adjustable from 15 to 70 nm; ✓ Used to prepare nanoparticles as doxorubicin injection delivery vehicles—prolonging drug release for over 6 days;	[74]
	✓ Tested as polymeric nanoparticles at contact lenses containing natamycin—use of nanoparticles (NPs) contributed to increasing the efficiency of loading and elution of the drug from contact lenses and allowed to extend the supply of natamycin from 1 to 12 h;	[75]
	✓ Used in the production of SCL modified with natamycin Dex-b-PLA nanoparticles.	

**Table 2:** Non-biodegradable polymers.

Polymer	Characteristics	References
*Ethyl cellulose (EC.)* 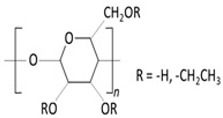	✓ Linear non-branched polysaccharide composed of glucose unit with β-(1, 4) glycoside linkage;	[76]
	✓ Hydroxyl end groups in the ethylcellulose structure replaced with ethyl groups at carbons 2, 3, or 6 of anhydrous glucose unit;	[77]
	✓ Non-ionic polymer; ✓ A wide range of stability in pH 3–11, biocompatible, approved by the FDA as “generally recognized as safe” substance for the oral, transdermal, and transmucosal routes;	[78]
	✓ Non-toxic polymer.	[79]
*Eudragit S-100* 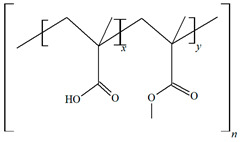	✓ Anionic copolymer of methacrylic acid and methacrylate in ratio ≈ 1:2; ✓ pH-sensitive;	[80]
	✓ Insoluble AT pH < 7; ✓ Biocompatible.	[81]
*Poly-HEMA* 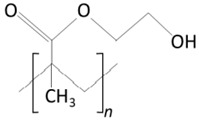	✓ Copolymer of 2-hydroxyethyl; ✓ Methacrylate;	[82]
	✓ A non-toxic polymer; ✓ Biomaterial; ✓ Biocompatible.	[83]

**Table 3:** Techniques used to test the mechanical parameters of contact lenses.

Parameter	The Technique Used to Test the Parameter	Description	Reference
Compression properties *Modulus of elasticity* *(Young’s modulus)*	Parallel plate compression (PPC)	It assesses the elastic recovery of contact lenses after deformation caused by the eyelid. An external force is applied to the lens via a fixture.	[91]
	Central load compression (CLC)	In this version, an external force is applied with a ball bearing.	
Toughness	Tensile tester instrument, Instron 1122 (Instron, Norwood, MA, USA)	The contact lens is subjected to tension to the critical point (fracture). A stress–strain curve is obtained, and the mechanical strength is determined.	[87]
*Stress relaxation (SR)*	ClearWave (Lumetrics Inc., Rochester, NY, USA) and OptiGauge II (Lumetrics Inc., New York, Rochester, USA)	The application of graded stress to a contact lens to maintain a specified strain as a function of time.	[92,93]
*Compression modulus*	Tensile tester instrument, Instron 1122 (Instron, Norwood, MA, USA)	The contact lens is subjected to tension to the critical point (fracture). A stress–strain curve is obtained, and the mechanical strength is determined.	[87]
*Glass-transition temperature*	Modulated differential scanning calorimetry; DSC 2920 (TA Instruments, New Castle, DE, USA), thermogravimetric analysis.	Alternating heating and cooling (dry and wet soft contact lenses) at a rate (2.5 °C/min), modulating with a sine wave.	[94]
*Elongation-at-break*	Instron 1122 tensile tester instrument (Instron, Norwood, MA, USA)	The contact lens is subjected to tension to the critical point (fracture). A stress–strain curve is obtained, and the mechanical strength is determined.	[87]
*Tensile strength*	Instron 1122 tensile tester instrument (Instron, Norwood, MA, USA)	The contact lens is subjected to tension to the critical point (fracture). A stress–strain curve is obtained, and the mechanical strength is determined.	[87]

**Table 4:** The polymer matrix’s influence on Cyclosporine A’s stability and release.

Polymer–CL Material	CyA Metabolites	Drug Loading Method	In Vivo and In Vitro	Reference
Non-biodegradable polymers				
Hydrogel CLs fabricated by cast moulding method using polypropylene lens form (60% *w*/*w* hydroxyethyl methyl acrylate (HEMA), 2% *w*/*w* methacrylic acid (MAA), 0.5% *w*/*w* ethylene glycol-dimethyl acrylate (EGDMA) and 1% *w*/*w* 1-vinyl-2-pyrrolidinone; 36.5% water)	-	pH-sensitive drug/Eudragit S99quasi-emulsion solvent diffusion; soaking for direct drug-loaded C.L.s.	In vivo, sustained release for up to 14 days.	[10]
Methoxy poly(ethylene glycol) (mPEG)-PLA with different weight ratios (40:60, 50:50, 60:40) with CyA-loaded micelles	-	Dissolving	The in vitro release profile study showed a sustained diffusion profile of CyA. Cyclosporine A micelles were stable for up to 10 days under long storage conditions.	[22]
Cellulose acetate phthalate-based pH-responsive polymer	-	The Cyclosporine A coating solution (CyA with poly(ethylene glycol) methyl ether methacrylate, HEMA, EGDMA, and azobisisobutyronitrile (AIBN) in appropriate proportions) was printed on the top mould and heated at 120 °C for 20 min.	CyA was stable in storage (4 °C, pH 5.4) for 90 days.	[11]
Intraocular lens implant–carrier PLGA	-	Encapsulation	The expected CyA release time is 12 weeks. In vitro tests showed that CyA concentration in aqueous humour was 146 mg/L at one-week post operation. Then, it gradually decreased to 0.15 mg/L at ten weeks of post operation.	[98]
-	Metabolite 7 and 10	-	The research aimed to separate metabolites 7 and 10 of CyA isolated from rabbit bile to determine the structure of CyA metabolites by mass spectrometric techniques.	[99]
-	The acidic metabolite of CyA with the n-methyl group of the Cyclosporine-specific nine-carbon amino acid #l has been oxidized to an α,β- unsaturated carboxylic acid functionality.	-	The study aimed to isolate the primary biliary metabolite of CyA from rabbit and human bile. The obtained metabolite was characterized using mass spectrometry and nuclear magnetic resonance spectrometry.	[100]
-	AM1, AM1c, AM4N, AM9, AM19, AM14N, AM49, AM1c9, Am4N9	-	The study aimed to determine selected metabolites of CyA in the tear fluid of patients using 2% CyA solution plus systemic steroids twice daily.	[101]
HEMA, MAA, glycerin, GMA, ethylene glycol dimethyl acrylate and initiator [2,2-Azobis (2,4-dimethyl valeronitrile)]	-	Supercritical fluid method on a porous silica carrier	Prepared contact lenses showed CyA release in the therapeutic range of up to 48 h. CyA was released to the surface in the rabbit eye for 48 h.	[102]
HEMA, EGDMA (10 mL) and TPO (Diphenyl(2,4,6-trimethyl benzoyl)phosphine oxide) Irgacure	-	Encapsulation of CyA/C-HA (cholesterol hyaluronate) micelles	In vitro drug release tests showed the possibility of administering CyA in therapeutic concentrations for more than 12 days.	[103]
1-DAY ACUVUE^®^ *(Etafilcon A, 58% water)* ACUVUE^®^ OASYS™ *(Senofilcon A, 38% water)* NIGHT&DAY™ *(Lotrafilcon A, 24% water)* O2OPTIX™ *(Lotrafilcon B, 33% water)* PureVision™ *(Balafilcon A, 36% water)*	-	The soaking/dip coating process	The research aimed to obtain CyA-modified contact lenses by soaking them in 10 mL of a 15 μg/mL CyA-PBS solution for seven days. Five types of CLs available on the market were examined. The ACUVUE OASYS lenses obtained the release profile closest to the zero profile. For 1-DAY ACUVUE^®^ lenses, the CyA supply was around 24 h, while the remaining lenses lasted more than seven days.	[9]
Biodegradable polymers				
Poly(lactide-co-glycolide) and PCL nanoparticles-loaded implants	-	Moulding and electrospinning technique	The in vitro release studies demonstrated sustained release for 30 to 60 days, with cell viability ranging from 77.4% to 99.0%. In vivo studies revealed that the selected implant formulation significantly accelerates healing.	[104]
Poly(lactide-co-glycolide) and PCL nanoparticles-loaded implants	-	Moulding and electrospinning technique	Results from a tissue distribution study revealed that CyA remained detectable in ocular tissues, including the cornea, sclera, and lens, even 90 days after application. Furthermore, efficacy studies demonstrated that using CyA-loaded fibre implant formulation led to a faster recovery, as evidenced by improved staining scores.	[105]
PLGA	-	Introducing CyA to the monomer mixture	The median survival time of untreated corneal allografts was 8.2 ± 1.48 days. The survival time for grafts treated with topical cyclosporine was 8.5 ± 1.50 days, while for polymer-only anterior chamber implants, it was 10.6 ± 1.90 days. Grafts treated with 1% cyclosporine drops had a median survival time of 11.4 ± 2.50 days, and those given subconjunctival cyclosporine–polymer had a survival time of 17 ± 3.05 days. Autografted rats showed a survival time of more than three months.	[106]
PLGA	-	Introducing CyA to the monomer mixture	In the initial 13-week in vitro tests, negligible amounts of CyA were observed to be released from the devices. It was concluded that the aqueous permeability in the polymeric matrix was insufficient to facilitate the release of CyA during this stage. Subsequently, between the 14th and 23rd week of the test, approximately 8.4% of CyA was released from the intraocular implants under sink conditions.	[107]
PLGA-PMMA	-	-	In vitro tests indicated that using CyA-PLGA-PMMA lenses did not result in any discernible toxic reactions in the intraocular tissues.	[108]

**Table 5:** Methods for improving the incorporation of CyA into polymeric materials.

Technique	Scheme	Description	Reference
CLs’ soaking technique assisted by the creation of a diffusion barrier (VE)	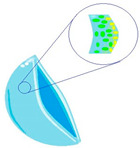	Using a hydrophobic vitamin E diffusion barrier extends drug delivery duration. The literature suggests incorporating vitamin E into silicone hydrogel (SiH) contact lenses with CyA prolongs elution for two weeks to a month.	[9]
		The vitamin E barrier extends CyA release from Hilafilcon B contact lenses from 150 to 300 min.	[109]
Cyclodextrin-based CL	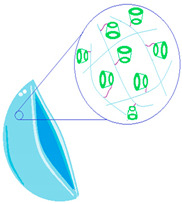	The study aimed to determine the optimal drug concentration among four formulations with different CyA and α cyclodextrin (αCDs) compounds, promoting the maximum and lowest corneal permeability (up to 750 mg/mL in aqueous solutions). Three formulations with the following compositions were tested: F1(15% γCD); F5 (10% γCD + 4% αCD); F7 (5% αCD) and the corresponding solid CyA fractions (%). Measurements were carried out over three months at different temperatures (5 °C, 25 °C and 40 °C). CyA was stable in all three formulations at all temperatures tested.	[110]
		The study established that applying one drop of a solution with a concentration of 0.025% *w*/*v* CyA in 40 mg/mL α-CD solution in four doses every 2 h to the eye of a rabbit allows for obtaining a concentration on the corneal surface that is 5–10 times higher than the concentration of the drug after applying conventional ointment with a starting concentration of 10% *w*/*w*. It exceeded the therapeutic dose.	[111]
		The research aimed to develop supramolecular CyA complexes (binary and ternary) based on sulfobutylether-β-cyclodextrin (SBE-β-CD). Complexation enabled an increase in solubility close to 21-fold, with a 4-fold increase in the penetration of the active substance through the cornea.	[12]
		CyA inclusion complex (2-hydroxypropyl)-β-cyclodextrin (HP-β-CD) with tocopheryl polyethylene glycol succinate (TPGS) was prepared by the freeze-drying method. The phase solubility results showed a high stability constant for tested samples.	[112]
Molecular imprinting	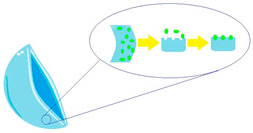	The study aimed to determine the total concentration of CyA and CyA metabolites such as AM1, AM9, and AM4N in human blood.	[113]
**Incorporation of colloidal nanoparticles**			
Polymeric nanoparticles	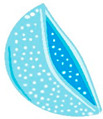	The nanoparticle-laden contact lenses with a 1:1 (drug: Eudragit) weight ratio were characterized by the most extended CyA supply of 156 h. The in vivo study on determining drug concentration in rabbit tear fluid showed a sustained stable release for up to 14 days.	[10]
Microemulsions	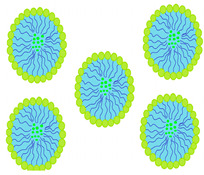	The research aimed to design a novel microemulsion in situ electrolyte-triggered gelling system for CyA. A microemulsion was prepared based on castor oil, Solutol HS 15, glycerol, and water and dispersed in a Kelcogel^®^ solution. The test results showed that 32 h after application, CyA concentrations delivered by the microemulsion were within the therapeutic window.	[114]
	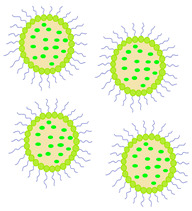	Microemulsion and surfactant-laden pHEMA hydrogels—Incorporation of CyA-laden microemulsions or surfactants with CyA directly into pHEMA pre-polymerization mixture. The research aimed to obtain Brij surfactant-laden p-HEMA gels releasing CyA in a typical manner for prolonged CyA supply. DDS obtained in tests possessed suitable mechanical and optical properties for ophthalmological applications.	[115]
		The research aimed to obtain a stable microemulsion using poly (2-hydroxyethyl methacrylate) (p-HEMA) hydrogels containing microemulsions or micelles of Brij 97 for the drug delivery of CyA. Results show that the surfactant and microemulsion-laden gels can provide an adequate and stable drug supply in therapeutic doses for about 20 days.	[16]
		The study examined the effects of surfactant chain lengths [sodium caprylate (C8), Tween 20 (C12), Tween 80 (C18)] and molecular weight of block copolymers [Pluronic F68 and Pluronic F127] on microemulsion stability and CyA release time from HEMA lenses. It has been shown that the stability of the microemulsion increases with an increase in the carbon chain lengths of surfactants and the molecular weight of pluronics. The most prolonged CyA supply in the therapeutic range (in vitro, for PL-127-T80) lasted 24 days.	[15]
Micelles		In vitro, drug release tests from a cholesterol hyaluronate (C-HA) micelle-embedded contact lens revealed the stable, controlled delivery of CyA for more than 12 days.	[103]
		CyA micellar formulation retained good physicochemical and microbiological stability at two conservation conditions (5 °C and 25 °C). Observed concentrations were variable for 20 mg/mL units stored at 25 °C.	[116]
		In vivo, ocular distribution studies from micelles exhibited a 4.5-fold retention effect compared with 0.05% CyA emulsion. In vitro stability tests indicated that CyA-loaded micellar lyophilized powder was stable for at least three months.	[19]
		Aqueous micellar formulation of vitamin E (TPGS: alpha tocopheryl polyethylene glycol 1000 succinate) and poloxamer 407 (Pluronic^®^ F127) with CyA were tested (molar ratio 1:1). Micelles were stable against dilution.	[117]
		The study aimed to investigate the kinetics of CyA release from hyaluronic acid HA-contact lenses. Two types of micelles were tested. Pluronic^®^ F127 micelles showed greater stability (up to 14 days), sustained release (120 h), and improved outcomes compared to Tween^®^ 80 micelles (96 h release).	[21]
		This work aimed to develop a micellar formulation capable of solubilizing a considerable amount of Closporine. For this purpose, non-ionic amphiphilic polymers (tocopherol polyethylene glycol 1000 succinate (TPGS) and Solutol^®^ HS15) were used for micelle preparation.	[118]
		Results showed that TPGS micelles loaded with 5 mg/mL of Closporine A promoted drug retention on the ocular surface. The polymer micelles that were the subject of the research were characterized by greater stability than micelles formed by surfactants.	
		The research aimed to obtain lyophilized methoxy poly(ethylene glycol)-poly(lactide) (mPEG-PLA) polymer micelles. The results showed a sustained release of CyA from the micelles and the stabilizer mPEG2000 could increase the in vitro stability of the lyophilized CyA-loaded mPEG-PLA micelle lyophilized formulations which were stable for ten days at temperatures from 40 to 60 degrees Celsius both with and without exposure to light.	[22]
		The research aimed to create stable nano micelles as a source of CyA. For this purpose, two non-ionic surfactants (d-α-tocopherol polyethylene glycol succinate, VE-TPGS) were used. The obtained products were characterized by a high CyA-EE (entrapment efficiency) content, and their parameters were comparable to those of the commercial Ikervis^®^ emulsion.	[119]
		Nanomicelles were stable at temperatures of 4 and 20 degrees Celsius throughout the entire measurement period of 60 days.	
Surfactants	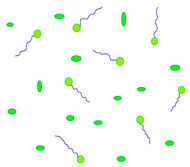	The study examined the impact of surfactant Brij 98 on CyA release from p-HEMA lenses. The developed models predicted a correlation between an increase in surfactant content (fourfold increase) and a decrease in the percentage of CyA and Brij 98 release over time (twofold). The indicated relationship allowed for the prolonged release of CyA from the lenses.	[120]
		The objective of this study was to determine Cyclosporine A (CyA) levels in ocular tissues and fluids after application of poly-3-caprolactone (PCL)/benzalkonium chloride (BKC) nanospheres and hyaluronic acid (HA) coated PCL/BKC nanospheres onto healthy rabbit corneas. Studies have shown that CyA-loaded PCL/BKC and HA-coated PCL/BKC nanospheres can achieve concentrations 10–15-fold higher than that obtained after applying the drug in solution in castor oil.	[121]
Liposomes	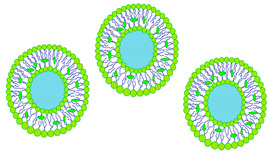	Fabricated CyA-loaded liposomes (CyA-Lips) were stable for 21 days. The optimized formulation for CyA-Lips was obtained with a ratio of egg yolk (Lecithin) to cholesterol set as 15 and a ratio of egg yolk (Lecithin) to cholesterol set as 2. The particle size of CyA Lips was 129.2 nm after optimization.	[24]
Transferosomes (type of liposomes)		The research aimed to use transferosomes as potential CyA carriers in ocular delivery. For this purpose, linoleic acid and its effect on the stability of the transferosomes were analyzed. Additionally, surfactants such as Span^®^ 80 and Tween^®^ 80 were examined for their impact on transferosome flexibility and toxicity to ocular cells as edge activators. The linoleic acid improved the stability of the transferosomes. The obtained transferosomes were stable for investigation of 4 months at −20 °C.	[122]

**Table 6:** Stability tests of the active substances.

Type of Test	Brief Description	Reference
Physical	It concerns several physical properties, including appearance, palatability, uniformity, and dissolution.	[131,132]
Chemical	Chemical tests to determine the durability and safety of a drug by monitoring toxic degradation products.	[133]
Microbiological	Identification of formulation parameters that prevent degradation of the active substance and ensure its microbiological safety.	[134,135]
Therapeutic	Activities focused on ensuring the stability of medical preparations and averting adverse alterations in formulations that could compromise their therapeutic efficacy.	[136]
Toxicological	Process steps and parameters to ensure the safety of the therapy and the formulation/system used in the context of toxicological safety (possible degradation and byproducts)	[137,138]

**Table 7:** Techniques used to test the stability of Cyclosporine A [141].

Technique	Formulation	Reference
High-performance Liquid chromatography (HPLC)	Liquid-filled capsules	[142]
	Oral solution	[143]
	In bulk drug and ophthalmic formulations	[144]
	Intravenous CyA preparations stored in non-PVC containers	[145]
	CyA ointments	[146]
	Sandimmun^®^ with $MgSO_{4}$ in 5% dextrose injection	[147]
	CyA 1% in artificial tears (Tears Plus—polyvinyl alcohol 1.4% and Povidone 0.6%)	[148]
	CyA diluted to 0.2 or 2.5 mg/mL with a 0.9% sodium chloride injection or 5% dextrose injection and stored in polypropylene–polyolefin containers or polypropylene syringes.	[149]
	CyA inserts prepared using hydroxypropyl methylcellulose	[150]
	lyophilized CyA-loaded polymeric micelles	[22]
	CyA as the bulk drug and in formulations (cationic nanoemulsion and ophthalmic drop formulation) in degradation terms	[144]
Mass spectrometry (MS)	Ophthalmic formulations (1%): Sandimmun^®^ and Novel ethanol-free formulation (CSA)	[151]
Ultraviolet–visible (UV–VIS) spectrophotometry	Cyclosporine-loaded Eudragit RL100 nanoparticles with 2% PVA	[152]
Solid-phase extraction (SPE)	CyA in a patented nanocarrier (Lipidot^®^)	[153]
High-performance Liquid chromatography–mass Spectrometry (HPLC-MS)	Sample of whole blood	[154]
	In cat blood	[155]
	Water-soluble prodrug of CyA (UNIL088)	[156]
Cyclo–Tractm SP 125J Radioimmunoassay	In whole-blood samples	[157]
Size exclusion chromatography (SEC)	CyA loaded poly(D,L lactide–glycolide) carriers	[158]
X-ray diffraction; scanning electron microscopic (SEM)	Amorphous solid dispersion (SD) of CyA employing hydroxypropyl cellulose (HPC)	[159]

## Data Availability

The dataset is available on request from the authors.

## Abbreviations

AIBN	Azobisisobutyronitrile
ASE	Accelerated solvent extraction
BKC	Benzalkonium chloride
CD	Cyclodextrin
CE-MS	Capillary electrophoresis–mass spectrometry
C-HA	Cholesterol hyaluronate
CL	Contact lens
CLC	Central load compression
CT	Chitosan
CyA	Cyclosporine A
CyA-EE	CyA entrapment efficiency
DDS	Drug delivery system
Dex-b-PLA	Poly (D,L-lactide)-dextran
EC	Ethyl cellulose
EGDMA	Ethylene glycol-dimethyl acrylate
FDA	Food and Drug Administration
FS	Fibrin sealant
GC-MS	Gas chromatography-mass spectrometry
HA.	Hyaluronic acid
HEMA	Hydroxyethyl methyl acrylate
HPC	Hydroxypropyl cellulose
HPLC	High-performance liquid chromatography
HPLC-MS	High-performance liquid chromatography–mass spectrometry
HP-β-CD	(2-hydroxypropyl)-β-cyclodextrin
LC-FTIR	Liquid chromatography Fourier-transform infrared
LC-MS	Liquid chromatography–mass spectrometry
LC-NMR	Liquid chromatography–nuclear magnetic resonance
LPLC	Low-pressure liquid chromatography
MAA	Methacrylic acid
MANOVA	Multivariate analysis of variance
mPEG	Metoxy poly(ethylene glycol)
mPEG-PLA	Methoxy poly(ethylene glycol)-poly(lactide)
MS	Mass spectrometry
NMR	Nuclear magnetic resonance
NPs	Nanoparticles
PCL	Polycaprolactone
PCL	Poly-3-Caprolactone
PGA	Polyglycolic acid
PGT	Propoxylated Glycerol Triacrylate
PLA	Polylactic acid
PLGA	Polylactic glycolic acid
PLTF	Pre-Lens Tear Film
POLTF	Post-Lens Tear Film
Poly-HEMA	Poly-hydroxy ethyl methacrylate
PPC	Parallel plate compression
PRF	Platelet-rich fibrin
RB.	Retinoblastoma
SBE-β-CD	Sulfobutylether-β-cyclodextrin
SEC	Size exclusion chromatography
SEM	Scanning electron microscopic
SiH	Silicone hydrogel
SPE	Solid–phase extraction
SR.	Stress relaxation
TET	Tetracycline
TI.	Therapeutic index
TLC	Thin-layer chromatography
TPGS	Tocopherol polyethylene glycol succinate
TPO	Diphenyl(2,4,6-trimethyl benzoyl)phosphine Oxide
TPT	Topotecan
TSCL	Therapeutic soft contact lenses
UV–VIS	Ultraviolet–visible
VE	Vitamin E
VE-TPGS	d-α-tocopherol polyethylene glycol succinate

## References

[1] Lanier O.L., Manfre M.G., Bailey C., Liu Z., Sparks Z., Kulkarni S., Chauhan A. (2021). Review of Approaches for Increasing Ophthalmic Bioavailability for Eye Drop Formulations. AAPS PharmSciTech.

[2] González-Chomón C., Concheiro A., Alvarez-Lorenzo C. (2013). Soft Contact Lenses for Controlled Ocular Delivery: 50 Years in the Making. Ther. Deliv..

[3] Kim J., Peng C.C., Chauhan A. (2010). Extended Release of Dexamethasone from Silicone-Hydrogel Contact Lenses Containing Vitamin E. J. Control. Release.

[4] Peng C.C., Burke M.T., Carbia B.E., Plummer C., Chauhan A. (2012). Extended Drug Delivery by Contact Lenses for Glaucoma Therapy. J. Control. Release.

[5] Li C.C., Chauhan A. (2006). Modeling Ophthalmic Drug Delivery by Soaked Contact Lenses. Ind. Eng. Chem. Res..

[6] Langer R. (1983). Implantable Controlled Release Systems. Pharmacol. Ther..

[7] Xu J., Xue Y., Hu G., Lin T., Gou J., Yin T., He H., Zhang Y., Tang X. (2018). A Comprehensive Review on Contact Lens for Ophthalmic Drug Delivery. J. Control. Release.

[8] Peng C.C., Kim J., Chauhan A. (2010). Extended Delivery of Hydrophilic Drugs from Silicone-Hydrogel Contact Lenses Containing Vitamin E Diffusion Barriers. Biomaterials.

[9] Peng C.C., Chauhan A. (2011). Extended Cyclosporine Delivery by Silicone-Hydrogel Contact Lenses. J. Control. Release.

[10] Maulvi F.A., Choksi H.H., Desai A.R., Patel A.S., Ranch K.M., Vyas B.A., Shah D.O. (2017). PH Triggered Controlled Drug Delivery from Contact Lenses: Addressing the Challenges of Drug Leaching during Sterilization and Storage. Colloids Surf. B Biointerfaces.

[11] Kim J., Mondal H., Jin R., Yoon H.J., Kim H.J., Jee J.P., Yoon K.C. (2023). Cellulose Acetate Phthalate-Based PH-Responsive Cyclosporine A-Loaded Contact Lens for the Treatment of Dry Eye. Int. J. Mol. Sci..

[12] Chaudhari P., Birangal S., Mavlankar N., Pal A., Mallela L.S., Roy S., Kodoth A.K., Ghate V., Nampoothiri M., Lewis S.A. (2022). Oil-Free Eye Drops Containing Cyclosporine A/Cyclodextrin/PVA Supramolecular Complex as a Treatment Modality for Dry Eye Disease. Carbohydr. Polym..

[13] Başaran E., Yenilmez E., Berkman M.S., Büyükköroǧlu G., Yazan Y. (2014). Chitosan Nanoparticles for Ocular Delivery of Cyclosporine A. J. Microencapsul..

[14] Wagh V.D., Apar D.U. (2014). Cyclosporine A Loaded PLGA Nanoparticles for Dry Eye Disease: In Vitro Characterization Studies. J. Nanotechnol..

[15] Maulvi F.A., Desai A.R., Choksi H.H., Patil R.J., Ranch K.M., Vyas B.A., Shah D.O. (2017). Effect of Surfactant Chain Length on Drug Release Kinetics from Microemulsion-Laden Contact Lenses. Int. J. Pharm..

[16] Kapoor Y., Chauhan A. (2008). Ophthalmic Delivery of Cyclosporine A from Brij-97 Microemulsion and Surfactant-Laden p-HEMA Hydrogels. Int. J. Pharm..

[17] Shen J., Liu X., Zhou M., Liu H. (2018). Novel Nanoemulsion Formulation for Ocular Drug Delivery for Cataract Therapeutics. J. Biomater. Tissue Eng..

[18] Terreni E., Chetoni P., Tampucci S., Burgalassi S., Al-Kinani A.A., Alany R.G., Monti D. (2020). Assembling Surfactants-Mucoadhesive Polymer Nanomicelles (ASMP-Nano) for Ocular Delivery of Cyclosporine-A. Pharmaceutics.

[19] Yu Y., Chen D., Li Y., Yang W., Tu J., Shen Y. (2018). Improving the Topical Ocular Pharmacokinetics of Lyophilized Cyclosporine A-Loaded Micelles: Formulation, In Vitro and In Vivo Studies. Drug Deliv..

[20] Mandal A., Gote V., Pal D., Ogundele A., Mitra A.K. (2019). Ocular Pharmacokinetics of a Topical Ophthalmic Nanomicellar Solution of Cyclosporine (Cequa^®^) for Dry Eye Disease. Pharm. Res..

[21] Maulvi F.A., Parmar M.B., Shetty K.H., Patel A.R., Desai B.V., Vyas B.A., Desai D.T., Kalaiselvan P., Masoudi S., Shah D.O. (2024). Role of Micelle Dynamics in Enhancing Cyclosporine Uptake in Hyaluronic Acid-Contact Lenses for Improved Critical Lens Properties in Dry Eye Management. Colloids Surf. A Physicochem. Eng. Asp..

[22] Shen Y., Yu Y., Chaurasiya B., Li X., Xu Y., Webster T.J., Tu J., Sun R. (2018). Stability, Safety, and Transcorneal Mechanistic Studies of Ophthalmic Lyophilized Cyclosporine-Loaded Polymeric Micelles. Int. J. Nanomed..

[23] Hwang S.-J., Karn P.R., Kim H., Kang H., Sun B.K., Jin S.-E. (2014). Supercritical Fluid-Mediated Liposomes Containing Cyclosporin A for the Treatment of Dry Eye Syndrome in a Rabbit Model: Comparative Study with the Conventional Cyclosporin A Emulsion. Int. J. Nanomed..

[24] Li Y., Guan Q., Xu J., Zhang H., Liu S., Ding Z., Wang Q., Wang Z., Liu M., Zhao Y. (2023). Comparative Study of Cyclosporine A Liposomes and Emulsions for Ophthalmic Drug Delivery: Process Optimization through Response Surface Methodology (RSM) and Biocompatibility Evaluation. Colloids Surf. B Biointerfaces.

[25] Gote V., Sikder S., Sicotte J., Pal D. (2019). Ocular Drug Delivery: Present Innovations and Future Challenges. J. Pharmacol. Exp. Ther..

[26] Van Santvliet L., Ludwig A. (2004). Determinants of Eye Drop Size. Surv. Ophthalmol..

[27] King-Smith P.E., Fink B.A., Hill R.M., Koelling K.W., Tiffany J.M. (2004). The Thickness of the Tear Film. Curr. Eye Res..

[28] Achouri D., Alhanout K., Piccerelle P., Andrieu V. (2013). Recent Advances in Ocular Drug Delivery. Drug Dev. Ind. Pharm..

[29] Gause S., Hsu K.H., Shafor C., Dixon P., Powell K.C., Chauhan A. (2019). Mechanistic Modeling of Ophthalmic Drug Delivery to the Anterior Chamber by Eye Drops and Contact Lenses. Adv. Colloid Interface Sci..

[30] Lanier O.L., Christopher K.G., Macoon R.M., Yu Y., Sekar P., Chauhan A. (2020). Commercialization Challenges for Drug Eluting Contact Lenses. Expert Opin. Drug Deliv..

[31] McNamara N.A., Polse K.A., Bonanno J.A. (1998). Fluorophotometry in Contact Lens Research: The next Step. Optom. Vis. Sci..

[32] Rykowska I., Nowak I., Nowak R. (2021). Soft Contact Lenses as Drug Delivery Systems: A Review. Molecules.

[33] Liu C., Zhu S.J., Zhou Y., Wei Y.P., Pei Y.Y., Pei Y.-Y., Road Y., Box P.O. (2006). Enhancement of Dissolution of Cyclosporine A Using Solid Dispersions with Polyoxyethylene (40) Stearate. Pharmazie.

[34] Choi S.W., Kim J. (2018). Therapeutic Contact Lenses with Polymeric Vehicles for Ocular Drug Delivery: A Review. Materials.

[35] Cussler E.L. (1997). Diffusion—Mass Transfer in Fluid Systems.

[36] Nichols J.J., Ewen King-Smith P. (2003). The Effect of Eye Closure on the Post-Lens Tear Film Thickness during Silicone Hydrogel Contact Lens Wear. Cornea.

[37] Creech J.L., Chauhan A., Radke C.J. (2001). Dispersive Mixing in the Posterior Tear Film under a Soft Contact Lens. Ind. Eng. Chem. Res..

[38] Parikh J.K., Vallabhbhai S., Engineer C., Parikh J., Raval A. (2011). Review on Hydrolytic Degradation Behavior of Biodegradable Polymers from Controlled Drug Delivery System. Trends Biomater. Artif. Organs.

[39] Mcnamara N.A., Polse K.A., Brand R.J., Graham A.D., Chan J.S., Mckenney C.D. (1999). Tear Mixing under a Soft Contact Lens: Effects of Lens Diameter. Am. J. Ophthalmol..

[40] Singh K., Nair A.B., Kumar A., Kumria R. (2011). Novel Approaches in Formulation and Drug Delivery Using Contact Lenses. J. Basic Clin. Pharm..

[41] Li X., Cui Y., Lloyd A.W., Mikhalovsky S.V., Sandeman S.R., Howel C.A., Liewen L. (2008). Polymeric Hydrogels for Novel Contact Lens-Based Ophthalmic Drug Delivery Systems: A Review. Contact Lens Anterior Eye.

[42] Kumar A., Jha G. (2011). Drug Delivery through Soft Contact Lenses: An Introduction. Chron. Young Sci..

[43] Grassie N., Scott G. (1985). Polymer Degradation and Stabilization.

[44] Lee S.S., Hughes P., Ross A.D., Robinson M.R. (2010). Biodegradable Implants for Sustained Drug Release in the Eye. Pharm. Res..

[45] Swarbrick J., Boylan J.C. (1989). Biodegradable Polyester Polymers as Drug Carriers to Clinical Pharmacokinetics and Pharmacodynamics. Encyclopedia of Pharmaceutical Technology.

[46] Merkli A., Tabatabay C., Gurny R., Heller J. (1998). Biodegradable Polymers for the Controlled Release of Ocular Drugs. Prog. Polym. Sci..

[47] Fredenberg S., Wahlgren M., Reslow M., Axelsson A. (2011). The Mechanisms of Drug Release in Poly(Lactic-Co-Glycolic Acid)-Based Drug Delivery Systems—A Review. Int. J. Pharm..

[48] Gijpferich A. (1996). Mechanisms of Polymer Degradation and Erosion. Biomaterials.

[49] Hussain C.M., Thomas S. (2021). Handbook of Polymer and Ceramic Nanotechnology.

[50] Hashim Thiab H. (2020). The Evaluation of Bandage Soft Contact Lenses as a Primary Treatment for Traumatic Corneal Abrasions. Int. J. Clin. Exp. Ophthalmol..

[51] Jung H.J., Abou-Jaoude M., Carbia B.E., Plummer C., Chauhan A. (2013). Glaucoma Therapy by Extended Release of Timolol from Nanoparticle Loaded Silicone-Hydrogel Contact Lenses. J. Control. Release.

[52] Jung H.J., Chauhan A. (2012). Temperature Sensitive Contact Lenses for Triggered Ophthalmic Drug Delivery. Biomaterials.

[53] Dash M., Chiellini F., Ottenbrite R.M., Chiellini E. (2011). Chitosan—A Versatile Semi-Synthetic Polymer in Biomedical Applications. Prog. Polym. Sci..

[54] Suh J.-K.F., Matthew H.W.T. (2000). Application of Chitosan-Based Polysaccharide Biomaterials in Cartilage Tissue Engineering: A Review. Biomaterials.

[55] Hirano S. (1999). Chitin and Chitosan as Novel Biotechnological Materials. Polym. Int..

[56] Yi H., Wu L.Q., Bentley W.E., Ghodssi R., Rubloff G.W., Culver J.N., Payne G.F. (2005). Biofabrication with Chitosan. Biomacromolecules.

[57] Dennyson S.A., Salih A., Alam F., Elsherif M., Alqattan B., Khan A.A., Yetisen A.K., Butt H. (2021). Ophthalmic Sensors and Drug Delivery. ACS Sens..

[58] Rinaudo M. (2008). Main Properties and Current Applications of Some Polysaccharides as Biomaterials. Polym. Int..

[59] Mourya V.K., Inamdar N.N. (2008). Chitosan-Modifications and Applications: Opportunities Galore. React. Funct. Polym..

[60] Naskar S., Koutsu K., Sharma S. (2019). Chitosan-Based Nanoparticles as Drug Delivery Systems: A Review on Two Decades of Research. J. Drug Target..

[61] Jain R.A. (2000). The Manufacturing Techniques of Various Drug Loaded Biodegradable Poly(Lactide-Co-Glycolide) (PLGA) Devices. Biomaterials.

[62] Bouissou C., Rouse J.J., Price R., Van Der Walle C.F. (2006). The Influence of Surfactant on PLGA Microsphere Glass Transition and Water Sorption: Remodeling the Surface Morphology to Attenuate the Burst Release. Pharm. Res..

[63] Makadia H.K., Siegel S.J. (2011). Poly Lactic-Co-Glycolic Acid (PLGA) as Biodegradable Controlled Drug Delivery Carrier. Polymers.

[64] Perinelli D.R., Cespi M., Bonacucina G., Palmieri G.F. (2019). PEGylated Polylactide (PLA) and Poly (Lactic-Co-Glycolic Acid) (PLGA) Copolymers for the Design of Drug Delivery Systems. J. Pharm. Investig..

[65] Malafaya P.B., Silva G.A., Reis R.L. (2007). Natural-Origin Polymers as Carriers and Scaffolds for Biomolecules and Cell Delivery in Tissue Engineering Applications. Adv. Drug Deliv. Rev..

[66] Tsui J.Y., Dalgard C., Van Quill K.R., Lee L., Grossniklaus H.E., Edelhauser H.F., O’Brien J.M. (2008). Subconjunctival Topotecan in Fibrin Sealant in the Treatment of Transgenic Murine Retinoblastoma. Investig. Ophthalmol. Vis. Sci..

[67] Kumar T.R.S., Bai M.V., Krishnan L.K. (2004). A Freeze-Dried Fibrin Disc as a Biodegradable Drug Release Matrix. Biologicals.

[68] Miron R.J., Zhang Y. (2018). Autologous Liquid Platelet Rich Fibrin: A Novel Drug Delivery System. Acta Biomater..

[69] Cruysberg L.P.J., Nuijts R.M.M.A., Gilbert J.A., Geroski D.H., Hendrikse F., Edelhauser H.F. (2005). In Vitro Sustained Human Transscleral Drug Delivery of Fluorescein-Labeled Dexamethasone and Methotrexate with Fibrin Sealant. Curr. Eye Res..

[70] Christen M.O., Vercesi F. (2020). Polycaprolactone: How a Well-Known and Futuristic Polymer Has Become an Innovative Collagen-Stimulator in Esthetics. Clin. Cosmet. Investig. Dermatol..

[71] Bartnikowski M., Dargaville T.R., Ivanovski S., Hutmacher D.W. (2019). Degradation Mechanisms of Polycaprolactone in the Context of Chemistry, Geometry and Environment. Prog. Polym. Sci..

[72] Dias J.R., Sousa A., Augusto A., Bártolo P.J., Granja P.L. (2022). Electrospun Polycaprolactone (PCL) Degradation: An In Vitro and In Vivo Study. Polymers.

[73] Nasr F.H., Khoee S., Dehghan M.M., Chaleshtori S.S., Shafiee A. (2016). Preparation and Evaluation of Contact Lenses Embedded with Polycaprolactone-Based Nanoparticles for Ocular Drug Delivery. Biomacromolecules.

[74] Verma M.S., Liu S., Chen Y.Y., Meerasa A., Gu F.X. (2012). Size-Tunable Nanoparticles Composed of Dextran-b-Poly(D,L-Lactide) for Drug Delivery Applications. Nano Res..

[75] Phan C.M., Subbaraman L., Liu S., Gu F., Jones L. (2014). In Vitro Uptake and Release of Natamycin Dex -b- PLA Nanoparticles from Model Contact Lens Materials. J. Biomater. Sci. Polym. Ed..

[76] Ahmadi P., Jahanban-Esfahlan A., Ahmadi A., Tabibiazar M., Mohammadifar M. (2022). Development of Ethyl Cellulose-Based Formulations: A Perspective on the Novel Technical Methods. Food Rev. Int..

[77] Kaur K., Kumar P., Kush P. (2020). Amphotericin B Loaded Ethyl Cellulose Nanoparticles with Magnified Oral Bioavailability for Safe and Effective Treatment of Fungal Infection. Biomed. Pharmacother..

[78] Wasilewska K., Winnicka K. (2019). Ethylcellulose-a Pharmaceutical Excipient with Multidirectional Application in Drug Dosage Forms Development. Materials.

[79] Leitner S., Grijalvo S., Solans C., Eritja R., García-Celma M.J., Calderó G. (2020). Ethylcellulose Nanoparticles as a New “In Vitro” Transfection Tool for Antisense Oligonucleotide Delivery. Carbohydr. Polym..

[80] Obeidat W.M., Price J.C. (2006). Preparation and Evaluation of Eudragit S 100 Microspheres as PH-Sensitive Release Preparations for Piroxicam and Theophylline Using the Emulsion-Solvent Evaporation Method. J. Microencapsul..

[81] Rehman S., Ranjha N.M., Shoukat H., Madni A., Ahmad F., Raza M.R., Jameel Q.A., Majeed A., Ramzan N. (2021). Fabrication, Evaluation, in Vivo Pharmacokinetic and Toxicological Analysis of PH-Sensitive Eudragit S-100-Coated Hydrogel Beads: A Promising Strategy for Colon Targeting. AAPS PharmSciTech.

[82] Lu S., Anseth K.S. (1999). Photopolymerization of Multilaminated Poly(HEMA) Hydrogels for Controlled Release. J. Control. Release.

[83] Zare M., Bigham A., Zare M., Luo H., Rezvani Ghomi E., Ramakrishna S. (2021). Phema: An Overview for Biomedical Applications. Int. J. Mol. Sci..

[84] Iqbal A., Thomas R., Mahadevan R. (2021). Impact of Modulus of Elasticity of Silicone Hydrogel Contact Lenses on Meibomian Glands Morphology and Function. Clin. Exp. Optom..

[85] Tranoudis I., Efron N. (2004). Tensile Properties of Soft Contact Lens Materials. Contact Lens Anterior Eye.

[86] Galeski A. (2003). Strength and Toughness of Crystalline Polymer Systems. Prog. Polym. Sci..

[87] Lonnen J., Putt K.S., Kernick E.R., Lakkis C., May L., Pugh R.B. (2014). The Efficacy of Acanthamoeba Cyst Kill and Effects upon Contact Lenses of a Novel Ultraviolet Lens Disinfection System. Am. J. Ophthalmol..

[88] Duan X., Yuan H., Tang W., He J., Guan X. (2022). An Engineering Prediction Model for Stress Relaxation of Polymer Composites at Multiple Temperatures. Polymers.

[89] Bhamra T.S., Tighe B.J. (2017). Mechanical Properties of Contact Lenses: The Contribution of Measurement Techniques and Clinical Feedback to 50 Years of Materials Development. Contact Lens Anterior Eye.

[90] Bicerano J. (2002). Glass Transition Operational Definition. Encyclopedia of Polymer Science and Technology.

[91] Mutlu Z., Shams Es-haghi S., Cakmak M. (2019). Recent Trends in Advanced Contact Lenses. Adv. Healthc. Mater..

[92] Compertore D.C., Ignatovich F.V. (2018). Measurement Accuracy of a Stressed Contact Lens during Its Relaxation Period. Ophthalmic Technol..

[93] Kim E., Saha M., Ehrmann K. (2018). Mechanical Properties of Contact Lens Materials. Eye Contact Lens.

[94] Fornasiero F., Ung M., Radke C.J., Prausnitz J.M. (2005). Glass-Transition Temperatures for Soft-Contact-Lens Materials. Dependence on Water Content. Polymer.

[95] (2017). Ophthalmic Optics. Contact Lenses. Part 4: Physicochemical Properties of Contact Lens Materials.

[96] Hsu K.H., Carbia B.E., Plummer C., Chauhan A. (2015). Dual Drug Delivery from Vitamin e Loaded Contact Lenses for Glaucoma Therapy. Eur. J. Pharm. Biopharm..

[97] Peng C.C., Burke M.T., Chauhan A. (2012). Transport of Topical Anesthetics in Vitamin e Loaded Silicone Hydrogel Contact Lenses. Langmuir.

[98] Pei C., Xu Y., Jiang J.X., Cui L.J., Li L., Qin L. (2013). Application of Sustained Delivery Microsphere of Cyclosporine A for Preventing Posterior Capsular Opacification in Rabbits. Int. J. Ophthalmol..

[99] Hartman N.R., Jardinet I. (1986). Mass Spectrometric Analysis of Cyclosporine Metabolites. Biomed. Environ. Mass Spectrom..

[100] Hartman N.R., Trimble L.A., Vederas J.C., Jardine I. (1985). An Acid Metabolite of Cyclosporine A. Biochem. Biophys. Res. Commun..

[101] Phillips T.M., Chmielinska J.J. (1994). Immunoaffinity Capillary Electrophoretic Analysis of Cyclosporin in Tears. Biomed. Chromatogr..

[102] Choi J.H., Li Y., Jin R., Shrestha T., Choi J.S., Lee W.J., Moon M.J., Ju H.T., Choi W., Yoon K.C. (2019). The Efficiency of Cyclosporine A-Eluting Contact Lenses for the Treatment of Dry Eye. Curr. Eye Res..

[103] Mun J., Mok J.W., Jeong S., Cho S., Joo C.K., Hahn S.K. (2019). Drug-Eluting Contact Lens Containing Cyclosporine-Loaded Cholesterol-Hyaluronate Micelles for Dry Eye Syndrome. RSC Adv..

[104] Pehlivan S.B., Yavuz B., Çalamak S., Ulubayram K., Kaffashi A., Vural I., Çakmak H.B., Durgun M.E., Denkbaş E.B., Ünlü N. (2015). Preparation and in Vitro/in Vivo Evaluation of Cyclosporine A-Loaded Nanodecorated Ocular Implants for Subconjunctival Application. J. Pharm. Sci..

[105] Yavuz B., Bozdağ Pehlivan S., Kaffashi A., Çalamak S., Ulubayram K., Palaska E., Çakmak H.B., Ünlü N. (2016). In Vivo Tissue Distribution and Efficacy Studies for Cyclosporine A Loaded Nano-Decorated Subconjunctival Implants. Drug Deliv..

[106] Xie L., Shi W., Wang Z., Bei J., Wang S. (2001). Prolongation of Corneal Allograft Survival Using Cyclosporine in a Polylactide-Co-Glycolide Polymer. Cornea.

[107] Barbosa Saliba J., Cunha Junior A.D.S., Gomes E.C.D.L., Sander Mansur H., Rodrigues da Silva G. (2011). Development and Validation of a High Performance Liquid Chromatographic Method for Determination of Cyclosporine A from Biodegradable Intraocular Implants. Quim. Nova.

[108] Teng H., Sun J., Wen K., Han G., Tian F. (2022). Observation of Cyclosporin A: Sustained Release Intraocular Lens Implantation in Rabbit Eyes. Curr. Eye Res..

[109] Michałkiewicz O., Nowak I., Nowak R., Rykowska I. (2023). Daily Disposable Contact Lenses as a Platform for Ocular Drug Delivery of Cyclosporine A. Physicochem. Probl. Miner. Process..

[110] Jóhannsdóttir S., Jansook P., Stefánsson E., Loftsson T. (2015). Development of a Cyclodextrin-Based Aqueous Cyclosporin A Eye Drop Formulations. Int. J. Pharm..

[111] Cheeks L., Kaswan R.L., Green K. (1992). Influence of Vehicle and Anterior Chamber Protein Concentration on Cyclosporine Penetration through the Isolated Rabbit Cornea. Curr. Eye Res..

[112] Gilani S.J., Imam S.S., Ali S. (2024). Formulation and Evaluation of Multicomponent Inclusion Complex of Cyclosporine A. J. Incl. Phenom. Macrocycl. Chem..

[113] Senholdt M., Siemann M., Mosbach K., Andersson L.I. (1997). Determination of Cyclosporin A and Metabolites Total Concentration Using a Molecularly Imprinted Polymer Based Radioligand Binding Assay. Anal. Lett..

[114] Gan L., Gan Y., Zhu C., Zhang X., Zhu J. (2009). Novel Microemulsion in Situ Electrolyte-Triggered Gelling System for Ophthalmic Delivery of Lipophilic Cyclosporine A: In Vitro and in Vivo Results. Int. J. Pharm..

[115] Kapoor Y., Thomas J.C., Tan G., John V.T., Chauhan A. (2009). Surfactant-Laden Soft Contact Lenses for Extended Delivery of Ophthalmic Drugs. Biomaterials.

[116] Chennell P., Delaborde L., Wasiak M., Jouannet M., Feschet-Chassot E., Chiambaretta F., Sautou V. (2017). Stability of an Ophthalmic Micellar Formulation of Cyclosporine A in Unopened Multidose Eyedroppers and in Simulated Use Conditions. Eur. J. Pharm. Sci..

[117] Grimaudo M.A., Pescina S., Padula C., Santi P., Concheiro A., Alvarez-Lorenzo C., Nicoli S. (2018). Poloxamer 407/TPGS Mixed Micelles as Promising Carriers for Cyclosporine Ocular Delivery. Mol. Pharm..

[118] Ghezzi M., Ferraboschi I., Delledonne A., Pescina S., Padula C., Santi P., Sissa C., Terenziani F., Nicoli S. (2022). Cyclosporine-Loaded Micelles for Ocular Delivery: Investigating the Penetration Mechanisms. J. Control. Release.

[119] Terreni E., Zucchetti E., Tampucci S., Burgalassi S., Monti D., Chetoni P. (2021). Combination of Nanomicellar Technology and in Situ Gelling Polymer as Ocular Drug Delivery System (Odds) for Cyclosporine-A. Pharmaceutics.

[120] Kapoor Y., Chauhan A. (2008). Drug and Surfactant Transport in Cyclosporine A and Brij 98 Laden P-HEMA Hydrogels. J. Colloid Interface Sci..

[121] Yenice I., Mocan M.C., Palaska E., Bochot A., Bilensoy E., Vural I., Irkeç M., Atilla Hincal A. (2008). Hyaluronic Acid Coated Poly-ε-Caprolactone Nanospheres Deliver High Concentrations of Cyclosporine A into the Cornea. Exp. Eye Res..

[122] Uwaezuoke O., Du Toit L.C., Kumar P., Ally N., Choonara Y.E. (2022). Linoleic Acid-Based Transferosomes for Topical Ocular Delivery of Cyclosporine A. Pharmaceutics.

[123] Laracuente M.L., Yu M.H., McHugh K.J. (2020). Zero-Order Drug Delivery: State of the Art and Future Prospects. J. Control. Release.

[124] Acharya G., Park K. (2006). Mechanisms of Controlled Drug Release from Drug-Eluting Stents. Adv. Drug Deliv. Rev..

[125] Wise D.L., Langer R.S. (1984). Medical Applications of Controlled Release.

[126] Elmas A., Akyüz G., Bergal A., Andaç M., Andaç Ö. (2020). Mathematical Modelling of Drug Release. Res. Eng. Struct. Mater..

[127] Paarakh M.P., Jose P.A., Setty C.M., Christoper G.V. (2018). Release Kinetics—Concepts and Applications. Int. J. Pharm. Res. Technol..

[128] Grassi M., Grassi G. (2014). Application of Mathematical Modeling in Sustained Release Delivery Systems. Expert Opin. Drug Deliv..

[129] Dash S., Murthy P.N., Nath L., Chowdhury P. (2010). Kinetic Modeling on Drug Release from Controlled Drug Delivery Systems. Acta Pol. Pharm. Drug Res..

[130] Singhvi G., Singh M. (2011). Review: In-Vitro Drug Release Characterization Models. Int. J. Pharm. Stud. Res..

[131] Alhalaweh A., Alzghoul A., Mahlin D., Bergström C.A.S. (2015). Physical Stability of Drugs after Storage above and below the Glass Transition Temperature: Relationship to Glass-Forming Ability. Int. J. Pharm..

[132] Mahlin D., Bergström C.A.S. (2013). Early Drug Development Predictions of Glass-Forming Ability and Physical Stability of Drugs. Eur. J. Pharm. Sci..

[133] Yoshioka S., Stella V.J. (2002). Chemical Stability of Drug Substances. Stability of Drugs and Dosage Forms.

[134] González-González O., Ramirez I., Ramirez B., O’Connell P., Ballesteros M., Torrado J., Serrano D. (2022). Drug Stability: ICH versus Accelerated Predictive Stability Studies. Pharmaceutics.

[135] Dao H., Lakhani P., Police A., Kallakunta V., Ajjarapu S.S., Wu K.W., Ponkshe P., Repka M.A., Narasimha Murthy S. (2018). Microbial Stability of Pharmaceutical and Cosmetic Products. AAPS PharmSciTech.

[136] Heister E., Neves V., Lamprecht C., Ravi S., Silva P., Coley H.M., Mcfadden J. (2012). Drug Loading, Dispersion Stability, and Therapeutic Efficacy in Targeted Drug Delivery with Carbon Nanotubes. Carbon.

[137] Aldubayyan A.A., Castrignanò E., Elliott S., Abbate V. (2021). Stability of Synthetic Cathinones in Clinical and Forensic Toxicological Analysis—Where Are We Now?. Drug Test. Anal..

[138] Peters F.T. (2007). Stability of Analytes in Biosamples-an Important Issue in Clinical and Forensic Toxicology?. Anal. Bioanal. Chem..

[139] US Department of Health and Human Services, Food and Drug Administration, Center for Drug Evaluation and Research (CDER), Center for Biologics Evaluation and Research (CBER) (2015). Analytical Procedures and Methods Validation for Drugs and Biologics Guidance for Industry.

[140] Wolska E.K., Gajewska M., Sznitowska M. (2019). Trudności w Sporządzaniu Recepturowych Kropli Do Oczu z Cyklosporyną A. Farm. Pol..

[141] Sonawane J.K., Chavan S.M., Narkar I.P., Jale S.C., Tendulkar N.V., Jadhav V., Jain A. (2023). A Review of Stability Indicating Methods and Forced Degradation Studies. Int. J. Res. Publ. Rev..

[142] Xu X., Gupta A., Faustino P., Sathe P.M., Sayeed V.A., Khan M.A. (2013). Development and Validation of an HPLC Method for Dissolution and Stability Assay of Liquid-Filled Cyclosporine Capsule Drug Products. AAPS PharmSciTech.

[143] Kumar M., Singhal S.K., Singh A. (2001). Development and Validation of a Stability Indicating HPLC Assay Method for Cyclosporine in Cyclosporine Oral Solution USP. J. Pharm. Biomed. Anal..

[144] Deshmukh G.S., Sharma A.K. (2016). Pharmaceutical and Biological Evaluations Stability-Indicating Validated HPLC Method for Assay of Cyclosporine-A in Bulk Drug and Ophthalmic Formulations. Pharm. Biol. Eval..

[145] Li M. (2012). Stability Studies of Intravenous Cyclosporine Preparations Stored in Non-PVC Containers. Ph.D. Thesis.

[146] Dong Y., Qu H., Pavurala N., Wang J., Sekar V., Martinez M., Fahmy R., Ashraf M., Cruz C.N., Xu X. (2018). Formulation Characteristics and in Vitro Release Testing of Cyclosporine Ophthalmic Ointments. Int. J. Pharm..

[147] Nieforth K.A., Shea B.F., Sounev F., Scavone J.M. (1993). Stability of Cyclosporine with Magnesium Sulfate in 5% Dextrose Injection. Am. J. Health-Syst. Pharm..

[148] Fiscella R.G., Le H., Lam T.T., Labib S. (1996). Stability of Cyclosporine 1% in Artificial Tears. J. Ocul. Pharmacol. Ther..

[149] Li M., Forest J.M., Coursol C., Leclair G. (2011). Stability of Cyclosporine Solutions Stored in Polypropylene-Polyolefin Bags and Polypropylene Syringes. Am. J. Health-Syst. Pharm..

[150] Al-Saedi Z.H.F., Alzhrani R.M., Boddu S.H. (2016). Formulation and in Vitro Evaluation of Cyclosporine-A Inserts Prepared Using Hydroxypropyl Methylcellulose for Treating Dry Eye Disease. J. Ocul. Pharmacol. Ther..

[151] Ghiglioni D.G., Martino P.A., Bruschi G., Vitali D., Osnaghi S., Corti M.G., Beretta G. (2020). Stability and Safety Traits of Novel Cyclosporine a and Tacrolimus Ophthalmic Galenic Formulations Involved in Vernal Keratoconjunctivitis Treatment by a High-Resolution Mass Spectrometry Approach. Pharmaceutics.

[152] Gupta M.K., Mishra B., Prakash D., Rai S.K. (2009). Nanoparticulate Drug Delivery System of Cyclosporine. Int. J. Pharm. Pharm. Sci..

[153] Guillot A., Couffin A.C., Sejean X., Navarro F., Limberger M., Lehr C.M. (2015). Solid Phase Extraction as an Innovative Separation Method for Measuring Free and Entrapped Drug in Lipid Nanoparticles. Pharm. Res..

[154] Krnáč D., Reiffová K., Rolinski B. (2019). A New HPLC-MS/MS Method for Simultaneous Determination of Cyclosporine A, Tacrolimus, Sirolimus and Everolimus for Routine Therapeutic Drug Monitoring. J. Chromatogr. B Anal. Technol. Biomed. Life Sci..

[155] Mohamed R., Mercolini L., Cuennet-Cosandey S., Chavent J., Raggi M.A., Peyrou M. (2012). Validation of a Dried Blood Spot LC-MS/MS Approach for Cyclosporin A in Cat Blood: Comparison with a Classical Sample Preparation. J. Pharm. Biomed. Anal..

[156] Lallemand F., Perottet P., Felt-Baeyens O., Kloeti W., Philippoz F., Marfurt J., Besseghir K., Gurny R. (2005). A Water-Soluble Prodrug of Cyclosporine A for Ocular Application: A Stability Study. Eur. J. Pharm. Sci..

[157] Smith M.C., Sephel G.C. (1990). Long-Term in Vitro Stability of Cyclosporine in whole-Blood Samples. Clin. Chem..

[158] Chacon M., Molpeceres J., Berges L., Guzman M., Aberturas M.R. (1999). Stability and Freeze-Drying of Cyclosporine Loaded Poly(D,L Lactide-Glycolide) Carriers. Eur. J. Pharm. Sci..

[159] Sato H., Kawabata Y., Yuminoki K., Hashimoto N., Yamauchi Y., Ogawa K., Mizumoto T., Yamada S., Onoue S. (2012). Comparative Studies on Physicochemical Stability of Cyclosporine A-Loaded Amorphous Solid Dispersions. Int. J. Pharm..

[160] Maulvi F.A., Soni T.G., Shah D.O. (2016). A Review on Therapeutic Contact Lenses for Ocular Drug Delivery. Drug Deliv..

